# Frederick and Phrenology

**Published:** 1886-06

**Authors:** 


### FREDERICK AND PHRENOLOGY.

"Who," said King Frederick of Prussia, at a fete at Potsdam, which had attracted an unusually brilliant assemblage ; "who is that tall, bony old man with a head so full of character ?"

"Sire, it is Dr. Gall, the famous phrenologist."

"Ah, the phrenologist, eh ? Command him to dine with us to-morrow evening."

Next evening the king received the doctor affably, and they sat down to dinner with a dozen other convives, all blazing with decorations and uniforms, but awkward and constrained in manner and conversation.

"Doctor," said the king, at the conclusion of the repast, "pray let us see something of your skill. Examine these gentlemen's heads and tell me frankly what you think of their characters and dispositions from the indications afforded by their cranial development."

Gall arose and felt the head of his neighbor on the right, a stout, powerful man in a resplendent uniform, who had been addressed as "General."

"Speak frankly," said the king, seeing that the phrenologist seemed embarassed.

"His excellency," said Gall, "must be passsionately addicted to—to field sports and exciting pleasures ; he has a decided fancy for—for the battlefield and—"

The king smiled and pointed the phrenologist to his other neighbor, a small, alert, keen-eyed man in the diplomatic costume.

"This gentleman," said the doctor, "is—hum—is an expert in gymnastic exercises, an accomplished pedestrian ; very neat and graceful in all operations requiring manual dexterity—"

"Enough," said the king, rapping on the table, and as a score of soldiers entered, he continued, to the stupefaction of Dr. Gall, "remove these gentlemen to their cells. Allow me to put in plain language what you were reluctant to say : The general is a murderer under sentence, and your other neighbor is the most expert pickpocket and cutpurse in all Prussia, who has eluded capture on innumerable occasions. Examine your pockets."

The doctor did so, and found that his handkerchief, purse, watch, and snuff-box had disappeared. They were all returned to him next day, with a complimentary letter from the king, and a costly snuff-box bearing Frederick's portrait set in brilliants.

